# Progression of valve heart disease in a cohort of patients undergoing renal replacement therapy

**DOI:** 10.1590/2175-8239-JBN-2023-0036en

**Published:** 2023-11-13

**Authors:** Maria Eduarda Cavalcanti Tompson, José Arthur Viana de Oliveira Pimentel, Manuella de Amorim Silva, Marcelo Antônio Oliveira Santos-Veloso, Andrea Bezerra de Melo da Silveira Lordsleem, Sandro Gonçalves de Lima

**Affiliations:** 1 Hospital Memorial Jaboatão, Serviço de Terapia Intensiva, Recife, PE, Brazil.; 2 Hospital Alfa, Serviço de Clínica Médica, Recife, PE, Brasil.; 3 Universidade Federal de Pernambuco, Centro de Biociências, Recife, PE, Brazil.; 4 Centro Universitário Maurício de Nassau, Faculdade de Medicina, Recife, PE, Brazil.; 5 Universidade Federal de Pernambuco, Hospital das Clínicas, Serviço de Cardiologia, Recife, PE, Brazil.

**Keywords:** Heart Valve Diseases, Chronic Kidney Failure, Calcification, Valvopatias Cardíacas, Insuficiência Renal Crônica, Calcificação

## Abstract

**Introduction:**

Cardiovascular disease is an important cause of death among patients with chronic kidney disease (CKD). Valve calcification is a predictor of cardiovascular mortality and coronary artery disease.

**Objective:**

To assess heart valve disease frequency, associated factors, and progression in CKD patients.

**Methods:**

We conducted a retrospective study on 291 CKD patients at Hospital das Clínicas de Pernambuco. Inclusion criteria were age ≥ 18 with CKD and valve disease, while those on conservative management or with missing data were excluded. Clinical and laboratory variables were compared, and patients were categorized by dialysis duration (<5 years; 5–10 years; >10 years). Statistical tests, including chi-square, Fisher’s exact, ANOVA, and Kruskal-Wallis, were employed as needed. Simple and multivariate binary regression models were used to analyze valve disease associations with dialysis duration. Significance was defined as p < 0.05.

**Results:**

Mitral valve disease was present in 82.5% (240) of patients, followed by aortic valve disease (65.6%; 86). Over time, 106 (36.4%) patients developed valve disease. No significant association was found between aortic, pulmonary, mitral, or tricuspid valve disease and dialysis duration. Secondary hyperparathyroidism was the sole statistically significant factor for mitral valve disease in the regression model (OR 2.59 [95% CI: 1.09–6.18]; p = 0.031).

**Conclusion:**

CKD patients on renal replacement therapy exhibit a high frequency of valve disease, particularly mitral and aortic valve disease. However, no link was established between dialysis duration and valve disease occurrence or progression.

## Resumo

**Introdução:**

Doenças cardiovasculares são uma causa significativa de morte em pacientes com Doença Renal Crônica (DRC). A calcificação valvar é preditor de mortalidade cardiovascular e doença arterial coronariana.

**Objetivo:**

Avaliar a frequência, fatores associados e progressão de valvopatias em pacientes com DRC.

**Métodos:**

Coorte retrospectiva com 291 pacientes ambulatoriais no Hospital das Clínicas de Pernambuco. Inclusão: ≥18 anos com DRC e valvopatia; exclusão: tratamento conservador ou dados incompletos. Variáveis clínicas e laboratoriais foram comparadas e categorizadas por tempo de terapia dialítica (TTD): <5 anos, 5–10 anos, >10 anos. Foram aplicados os testes Qui-quadrado, exato de Fisher, ANOVA, Kruskal-Wallis. Associação entre valvopatia e TTD foi avaliada por regressão binária. Significância foi definida como p < 0,05.

**Resultados:**

A valvopatia mitral foi encontrada em 82,5% (240) dos casos, seguida da aórtica (65,6%; 86). Houve progressão da doença valvar em 106 (36,4%) pacientes. Não houve associação entre valvopatias aórtica, pulmonar, mitral ou tricúspide e TTD. Hiperparatireoidismo secundário foi a única variável explicativa significativa na regressão para valvopatia mitral (OR 2,59 [IC95%: 1,09–6,18]; p = 0,031).

**Conclusão:**

Encontramos alta frequência de valvopatias, especialmente mitral e aórtica, aem pacientes com DRC. Não houve associação entre TTD e valvopatia.

## Introduction

Chronic kidney disease (CKD) is a global public health problem associated with significant morbidity, mortality, and costs to the healthcare system. The prevalence of CKD in developed countries varies between 10–15%^1,2^. In the last decade, a significant increase in the prevalence of the disease has been observed in Brazil. In 2019, 139,691 patients were on dialysis in Brazil^3^.

Cardiovascular disease (CVD) is the main cause of death among patients with CKD. Mitral and aortic valve calcification stand out, with the latter described as a strong predictor of cardiovascular mortality^1,4,5^.

Aortic valve disease is more prevalent and severe in individuals with CKD when compared to the general population^6^. Mitral calcification occurs in as many as 40% of patients with CKD and severe involvement has been linked with increased all-cause mortality^7^.

Dialysis patients appear to have a higher incidence and faster progression of valve disease, especially in the aortic valve, due to sustained inflammatory state, oxidative stress, and mineral and bone disorder^8,9,10,11^. However, three large studies – Framingham OS, MESA and CRIC – failed to establish a direct association between kidney dysfunction and aortic stenosis^11,12,13^.

The main treatment available for valve disease is valve replacement surgery. However, the procedure has its limitations: mechanical valves require lifelong anticoagulation therapy, while biological valves have a lifespan of 10–15 years^14^.

This study aimed to look into the progression of heart valve disease in patients with CKD. Secondary endpoints included frequency of occurrence of heart valve disease and other associated factors.

## Methods

This retrospective cohort study included patients with CKD followed up at the heart and kidney disease outpatient clinic of the Hospital das Clínicas of the Federal University of Pernambuco between March 2008 and October 2021.

### Study Population

The study included patients with ages ≥18 years with CKD, as defined by the Kidney Disease: Improving Global Outcomes (KDIGO)^15^ initiative, diagnosed with valve disease based on transthoracic echocardiography (TTE). Patients with missing data on their medical records and individuals not undergoing renal replacement therapy (RRT) were excluded.

### Data Collection and Procedures

All medical records were reviewed and the following data were collected: anthropometric data, clinical data, and supplementary exams.

Duration of dialysis therapy (DDT) was defined as the difference, in years, between the date of the first dialysis session and last dialysis session, visit, transplantation, or death. The patients were categorized into three groups and compared based on DDT (>5 years; 5–10 years; >10 years).

TTE was performed at the first visit and repeated during follow-up as deemed necessary by the attending physician. The severity and mode of valve dysfunction (i.e., calcification, stenosis, and/or regurgitation) were assessed according to the guidelines of the American Society of Echocardiography^16^. When present, valve dysfunction was described as mild, moderate, or severe. Progression of valve disease was defined as an increase of at least one degree between follow-up examinations.

### Statistical Analysis

Data were analyzed descriptively using absolute frequencies and proportions. Pearson’s chi-square test or Fisher’s exact test was used to assess the association between categorical variables. The comparison of quantitative variables was performed using one-way ANOVA for variables with a normal distribution, or the Kruskal-Wallis test. Normality was assessed via the Shapiro-Wilk test.

The association between the occurrence of a given valve disease and DDT was confirmed using a binary logistic regression model, considering the presence of valve disease (individually) as a dependent variable and categorical DDT as an independent variable. In the case of a positive association, adjustments were made for baseline variables with statistically significant differences using a multivariate binary regression model. Odds ratios (OR) and 95% confidence intervals (95%CI) were presented as measures of association in the regression models.

Statistical analyses were performed on Statistical Package for the Social Sciences software version 20.0 (IBM, Armonk, NY, USA). The standard error used in every statistical test was 5%.

### Ethics

The study was evaluated and approved by the Institutional Research Ethics Committee, and given certificate 51098221.7.0000.8807. Data collection began only after approval was attained. Since this was a retrospective study, informed consent was not sought.

## Results

A total of 568 medical records were first reviewed, of which 347 were deemed eligible for inclusion; however, another 56 were excluded after further review. The final population consisted of a total of 291 patients aged 53.5 years on average, with a predominance of females (51.5%). The baseline clinical characteristics of the study population are described in Table 1.

**Table 1:** Baseline clinical characteristics of the study population stratified acc ording to duration of dialysis therapy (DDT)

	DDT < 5 years (n = 77)	DDT 5-10 years (n = 113)	DDT > 10 years (n = 101)	p-value
Age (years)	56.9 ± 13.9	52.7 ± 12.2	52.0 ± 11.5	0.001^†^
Female gender	49.3% (38)	51.3% (58)	53.4% (54)	0.85^‡^
RRT				
66.2% (51)	73.4% (83)	77.2% (78)	0.01^‡^	0,01^‡^
11.6% (9)	5.3% (6)	1.9% (2)	0.03^‡^	0,03^‡^
11.6% (9)	15.0% (17)	21.7% (22)	0.004^‡^	0,004^‡^
GFR (mL/min/1,73m^2^)				
9-60	2.5% (2)	2.6% (3)	0.0% (0)	0.22^‡^
59-30	0.0% (0)	3.5% (4)	3.9% (4)	
15-29	5.2% (4)	17% (2)	2.9% (3)	
< 15	49.3% (38)	53.9% (61)	67.3% (68)	
Comorbidities				
SAH	80.5% (62)	86.7% (98)	82.1% (83)	0.59^‡^
DM	51.9% (40)	15.0% (17)	9.9% (10)	< 0.001^‡^
Dyslipidemia	19.5% (15)	24.8% (28)	20.7% (21)	0.65^‡^
Smoking	40.3% (31)	31% (35)	96.0% (97)	0.33^‡^
Obesity	7.8% (6)	6.2% (7)	2.9% (3)	0.35^‡^
CAD	13% (10)	6.2% (7)	13.9% (14)	0.14^‡^
Stroke	7.8% (6)	11.5% (13)	11.9% (12)	0.63^‡^
PAD	6.5% (5)	8.0% (9)	4.0% (4)	0.47^‡^
Laboratory tests				
PTH (pg/mL)	726 ± 1086	1335 ± 924	1357 ± 928	0.002^§^
Phosphorus (mg/dL)	5.3 ± 1.4	5.6 ± 1.5	5.3 ± 1.4	0.37^†^
Total calcium (mg/dL)	9.5 ± 1.1	9.8 ± 1.3	10.0 ± 1.4	0.002^§^
Total cholesterol (mg/dL)	171.8 ± 62.0	187.2 ± 120.6	164.1 ± 50.4	0.40^§^
LDL cholesterol (mg/dL)	92.8 ± 50.0	94.0 ± 38.4	80.4 ± 37.1	0.18^§^
Triglycerides (mg/dL)	200.9 ± 232.3	164.8 ± 83.1	148.1 ± 98.9	0.24^§^
25-OH Vitamin D (ng/mL)	37.3 ± 11.8	27.8 ± 5.3	-	0.18^§^
Alkaline phosphatase (U/L)	362.4 ± 337.5	710 ± 1052	505.1 ± 661	0.54^§^
CRP (mg/L)	1.46 ± 1.49	4.14 ± 8.32	2.33 ± 3.59	0.21^§^

DDT: duration of dialysis therapy; RRT: renal replacement therapy; GFR: glomerular filtration rate; SAH: systemic arterial hypertension; DM: diabetes mellitus; CAD: coronary artery disease; CVA: cerebrovascular accident; PAD: peripheral arterial disease; PTH: parathyroid hormone; LDL: low-density lipoprotein; CRP: C-reactive protein.

† ANOVA;

‡ Chi-square;

§ Kruskal-Wallis.

Mitral valve disease was the most frequently observed condition (82.5%), followed by aortic valve disease (65.6%). During outpatient follow-up, progression of valve disease occurred in 106 (36.4%) patients. Five patients (1.7%) underwent mitral valve replacement surgery, of which four were given biological tissue valves. After surgery, two (50%) of the patients given biological tissue valves developed calcification. More than half (53.6%; 156) of the patients developed hyperparathyroidism secondary to CKD, with parathyroidectomies performed in 11.3% of the cases.

Univariate analyses found an association between higher DDT and development of mitral (74% vs. 83.2% vs. 88.1%; p = 0.048) and tricuspid (22.1% vs. 38.1% vs. 40.6%; p = 0.02) valve disease, and a higher incidence of secondary hyperparathyroidism (37.6% vs. 54.8% vs. 64.3%; p < 0.001), including the need for surgery.

Aortic (p = 0.09) and pulmonary valve (p = 0.56) involvement were not associated with DDT. Progression of valve disease (p = 0.95) and valve calcification (p = 0.92) occurred independently of DDT (Table 2).

**Tabela 2:** Comparison between frequencies of valve disease and associated factors according to duration of dialysis therapy (DDT>)

	DDT < 5 years (n = 77)	DDT 5-10 years (n = 113)	DDT > 10 years (n = 101)	p-value^†^
Presence of valve disease				
Mitral	74% (57)	83.2% (94)	88.1% (89)	0.048
Tricuspid	22.1% (17)	38.1% (43)	40.6% (41)	0.02
Aortic	67.5% (52)	58.4% (66)	72.3% (73)	0.09
Pulmonary	1.3% (1)	3.5% (4)	4.0% (4)	0.56
Valve disease progression	33,7% (26)	33.7% (26)	33.6% (38)	41.5% (42)
Symptomatic valve disease	13,3% (10)	13.3% (10)	15.6% (17)	20.6% (20)
Valve calcification	47,1% (33)	47.1% (33)	46.4% (45)	44.2% (42)
Hyperparathyroidism	37,6% (29)	37.6% (29)	54.8% (62)	64.3% (65)
Parathyroidectomy^‡^	6.8% (2/29)	16.1% (10/62)	50.7% (33/65)	<0.001

DDT: duration of dialysis therapy.

‡ Proportion of individuals undergoing parathyroidectomy relative to the total number of patients diagnosed with hyperparathyroidism.

† Chi-square.

**Figura 1.**
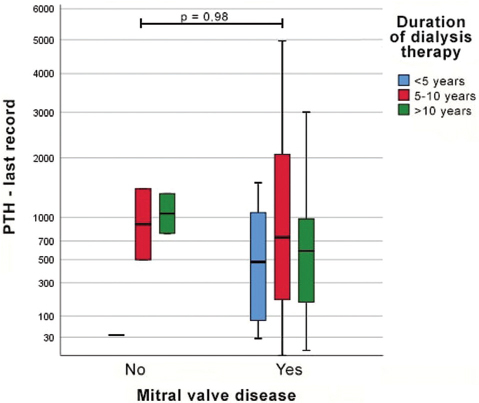
Comparison of serum PTH levels in relation to the presence of mitral valve disease and duration of dialysis therapy.

After adjustment for confounding variables, there was no association between occurrence of mitral or tricuspid valve disease and DDT. The presence of secondary hyperparathyroidism was the only explanatory variable that remained statistically significant in the regression model for mitral valve disease only (adjusted OR 2.59 [95% CI: 1.09–6.18]; p = 0.031) (Table 3). Nevertheless, there were no differences in median serum PTH levels associated with presence of valve disease (Figure 1).

**Tabela 3:** Simple binary logistic regression model adjusted for confounding variables

Outcome	Independent variable	Simple regression, OR (IC 95%)	p-value	Adjusted regression model, OR (IC 95%)	p-value
Mitral valve disease	DDT < 5 years	Reference	-	Reference	-
	DDT 5-10 years	1.73 (0.85-3.52)	0.12	1.83 (0.63-5.33)	0.26
	DDT > 10 years	2.60 (1.18-5.72)	0.018*	2.25 (0.66-7.67)	0.19
	RRT: hemodialysis	1.47 (0.67-3.24)	0.33	0.99 (0.23-4.24)	0.99
	Age	0.99 (0.97-1.02)	0.87	1.01 (0.98-1.04)	0.30
	DM	0.88 (0.47-1.65)	0.71	1.44 (0.48-4.24)	0.50
	Hyperparathyroidism	1.92 (1.05-3.49)	0.032*	2.59 (1.09-6.18)	0.031*
Tricuspid valve disease	DDT < 5 years	Reference	-	Reference	-
	DDT 5-10 years	2.16 (1.12-4.19)	0.021*	1.37 (0.55-3.36)	0.49
	DDT > 10 years	2.41 (1.23-4.70)	0.010*	1.02 (0.39-2.65)	0.96
	RRT: hemodialysis	1.70 (0.83-3.47)	0.14	6.66 (0.82-54.1)	0.07
	Age	1.00 (0.98-1.02)	0.88	1.00 (0.98-1.03)	0.48
	DM	0.63 (0.36-1.08)	0.09	0.56 (0.24-1.31)	0.18
	Hyperparathyroidism	1.84 (1.10-3.09)	0.019*	1.86 (0.89-3.87)	0.09

OR: odds ratio, 95% CI: 95% confidence interval; DDT: duration of dialysis therapy; RRT: renal replacement therapy; DM: diabetes mellitus.

* p-value < 0,05.

PTH: Serum parathyroid hormone (pg/mL); p-value calculated by the Mann-Whitney test.

## Discussion

Mitral and aortic valve disease are present in 30% and 15% of the patients with CKD, respectively. Other heart valves may be affected, although not as commonly^17–19^. In patients with CKD, the prevalence of mitral valve disease varies between 25% and 59%^18^. In our study, the frequency of valve disease was similar to that found in the literature, with mitral valve involvement being the most frequent.

Several factors may predispose patients with CKD to developing valve disease. Hypertension, diabetes, dyslipidemia and age have been related to valve calcification and atherosclerosis, suggesting that systemic atherosclerosis may play a role in the pathophysiology of calcification in individuals with CKD^20^. We analyzed the association between valve calcification in general or calcification in each specific heart valve and DDT, serum cholesterol levels, triglycerides levels, age, and presence of comorbid conditions, and found that only hyperparathyroidism was a predictor of mitral valve disease.

Several studies correlated valve calcification and progression of valve disease with phosphorus and calcium deposition^18,21–23^. Current studies also suggest a role for inflammation and lipid deposition in calcification^21,23,24^. Although we observed a high frequency of calcium, phosphorus and lipid level disorders in our population, we found no association between these variables and valve calcification. This is in agreement with the findings published by Ikee et al.^21^ and Cui et al.^22^, in which there was no association between mitral valve calcification and changes in bone mineral metabolism.

In our study, the presence of secondary hyperparathyroidism seemed to be a predictor of mitral valve disease. Nevertheless, serum PTH levels did not differ between patients with and without mitral valve disease, even when stratified by DDT. A possible explanation is the fact that more than half (50.7%) of the patients on dialysis for over 10 years had undergone parathyroidectomies to decrease serum PTH levels.

Hyperparathyroidism secondary to CKD has been previously described as an independent predictor of aortic valve stenosis^17^, while elevated PTH levels have been associated with severe aortic valve disease^25^. Presence of osteoblast-like cells, hydroxyapatite crystals, and overexpression of specific bone tissue proteins have been described in calcified valves^26^. These findings reinforce the relevance of the mechanism of calcification and cardiovascular degeneration, which are fundamentally related to the disruption of the calcium-phosphorus homeostasis, pathological bone remodeling, and reduction of calcification inhibitors^4^.

The pro-calcification and inflammatory state of CKD seems to accelerate the development of valve disease and the deterioration of biological tissue valves^19^. In our study, we found no association between valve disease progression and DDT. Two of the four patients given biological tissue valves developed calcification.

In addition to serum PTH levels, the state of hyperparathyroidism depends on a combination of clinical factors, such as: changes in calcium-phosphorus metabolism, vitamin D activity, nutritional and inflammatory status. Secondary hyperparathyroidism in CKD may also occur due to rickets, malabsorption, and pseudohypoparathyroidism^27^.

Similarly, premature cardiovascular calcification syndrome in CKD depends on the state of secondary hyperparathyroidism, hyperphosphatemia, bone mineral disease, and uremia-related cardiovascular changes. RRT affects various cardiovascular risk factors differently, however it is speculative how these changes translate into different levels of valve calcification^28^.

This study has its limitations. It is a retrospective study carried out at a single center based on convenience sampling; data collection was affected negatively by the lack of standardization of medical records; echocardiograms were performed by different operators, which may have generated inconsistencies.

Despite these limitations, our study is valuable in that it looked into patients with CKD with pulmonary and tricuspid valve disease, subtypes studied less often in the literature.

## Conclusion

We found a high frequency of valve disease in patients with CKD on RRT, involving particularly the mitral and aortic valves. No association was found between DDT, occurrence and/or progression of valve disease; only secondary hyperparathyroidism was associated with occurrence of mitral valve disease.
